# Numerical investigation of effects of incisor angle on production of sibilant /s/

**DOI:** 10.1038/s41598-021-96173-2

**Published:** 2021-08-18

**Authors:** HsuehJui Lu, Tsukasa Yoshinaga, ChungGang Li, Kazunori Nozaki, Akiyoshi Iida, Makoto Tsubokura

**Affiliations:** 1 Computational Fluid Dynamics Laboratory, Department of Computational Science, Graduate School of System Informatics, Kobe University, 1-1 Rokkodai, Nada-ku, Kobe, 657-8501 Japan; 2 Complex Phenomena Unified Simulation Research Team, RIKEN, Advanced Institute for Computational Science, Kobe, 650-0047 Japan; 3 Toyohashi University of Technology, 1-1 Hibarigaoka, Tempaku-cho, Toyohashi, Aichi 441-8580 Japan; 4 Osaka University Dental Hospital, 1-1 Yamadaoka, Suita, Osaka 565-0871 Japan

**Keywords:** Fluid dynamics, Computational biophysics

## Abstract

The effects of the inclination angle of the incisor on the speech production of the fricative consonant /s/ was investigated using an implicit compressible flow solver. The hierarchical structure grid was applied to reduce the grid generation time for the vocal tract geometry. The airflow and sound during the pronunciation of /s/ were simulated using the adaptively switched time stepping scheme, and the angle of the incisor in the vocal tract was changed from normal position up to 30°. The results showed that increasing the incisor angle affected the flow configuration and moved the location of the high turbulence intensity region thereby decreased the amplitudes of the sound in the frequency range from 8 to 12 kHz. Performing the Fourier transform on the velocity fluctuation, we found that the position of large magnitudes of the velocity at 10 kHz shifted toward the lip outlet when the incisor angle was increased. In addition, separate acoustic simulations showed that the shift in the potential sound source position decreased the far-field sound amplitudes above 8 kHz. These results provide the underlying insights necessary to design dental prostheses for the production of sibilant fricatives.

## Introduction

Fricative consonants are speech sounds that are produced via turbulent flow in the vocal tract. It is known that the central incisor position and angle affect speech production, especially that of the fricative consonant /s/ ^1–5^. During the articulation of /s/, a constricted flow channel (sibilant groove) is formed between the tongue tip and the upper incisors. Because the /s/ sound is generated by the turbulent jet flow leaving the constriction^6^, inaccurate formation of the constriction results in speech production difficulties for /s/.

Runte et al.^1^ constructed a maxillary denture, and the inclination angle of the central incisor was changed within the range of − 30° to + 30° to investigate the influence of the angle on the production of /s/. Meanwhile, the jaw movement during the phonation of /s/ was measured to observe the closest oral tract space with the horizontal and vertical overlap of the incisors^7^. Hamlet et al.^8^ measured the tongue movement with a contact sensor and showed the effects of dental prostheses on the timing and duration of the constriction formation of sibilant fricatives. However, because the /s/ sound is generated by jet flow in the oral tract, the detailed mechanisms of the effects of dental prostheses on the observed sound changes are unclear.

The production mechanisms in the absence of articulatory dysfunction have been investigated by modeling the vocal tract geometry. Shadle^9^ proposed a simplified vocal tract model for fricative consonants and investigated the effects of teeth-like obstacle positions on the generated sounds. In addition, numerical flow simulations were applied to a simplified model and the effects of geometrical differences on the turbulent jet flow and its sound sources were examined^10,11^.

To further investigate the airflow associated with the production of /s/ and its source characteristics in an actual oral tract, a vocal tract replica was constructed from computational tomography (CT) images and the flow and sound generated in the replica were measured using a microphone and an anemometer^12^. A numerical flow simulation of a realistic vocal tract geometry revealed that the aeroacoustic sound source is located near the upper and lower incisor surfaces^13^.

Recently, both turbulent flow for /s/ and the sound generated by the vocal tract geometry were predicted via numerical simulations using high-performance computing resources^14–17^. These simulations revealed that the sound source is located downstream of the upper and lower incisors and that the acoustic characteristics of /s/ are formed by the geometry downstream of the constriction. However, the effects of geometrical differences resulting from dental prosthesis, e.g., the incisor positions and angles, on the flow and sound generation are still unclear.

Therefore, in this study, we conducted numerical flow simulations of the vocal tract geometry of /s/ at different incisor angles to examine the origin of sound changes using the inclination angles of the incisor reported by Runte^1^. To explore the cause of the sound changes, both the airflow for /s/ and the sound in the vocal tract geometry were predicted using numerical simulations solving the three-dimensional compressible Navier–Stokes equation^18–20^. One difficulty with numerical flow simulations is maintaining high-quality computational grids for the complex flow channel in the vocal tract geometry. To reduce the grid generation time for the vocal tract geometry, we applied the hierarchical structure grid^21^ in the simulations. By further developing the proposed methodology, this simulation technology will enable us to predict the effects of dental prostheses on the production of sibilant fricatives for patients prior to prosthetic surgery.

## Methods

### Vocal tract geometry

To simulate the phenomena involved in the pronunciation of /s/, the geometry of a vocal tract replica was constructed from CT images^12^. The subject was a 32-year-old Japanese male who self-reported no speech disorders with a normal dentition of angle class I, without inter-teeth spacing. The CT images were taken while the subject sustained the pronunciation of /s/ for 9.6 s without vowel context, and the image resolution was 0.1 × 0.1 × 0.1 mm^3^. The surfaces of the vocal tract geometry were extracted based on brightness values using the software itk-SNAP^22^. Since the resolution of the CT scan is 0.1 mm and the tips of the incisors were slightly smoothed through the vocal tract extraction from the CT scan, we have confirmed that the extracted vocal tract geometry reproduced the subject’s pronunciation of /s/ up to 14 kHz by constructing an oral replica^12^. The ethics committee of the graduate school of Osaka University certified this study (H26-E39).

**Figure 1:**
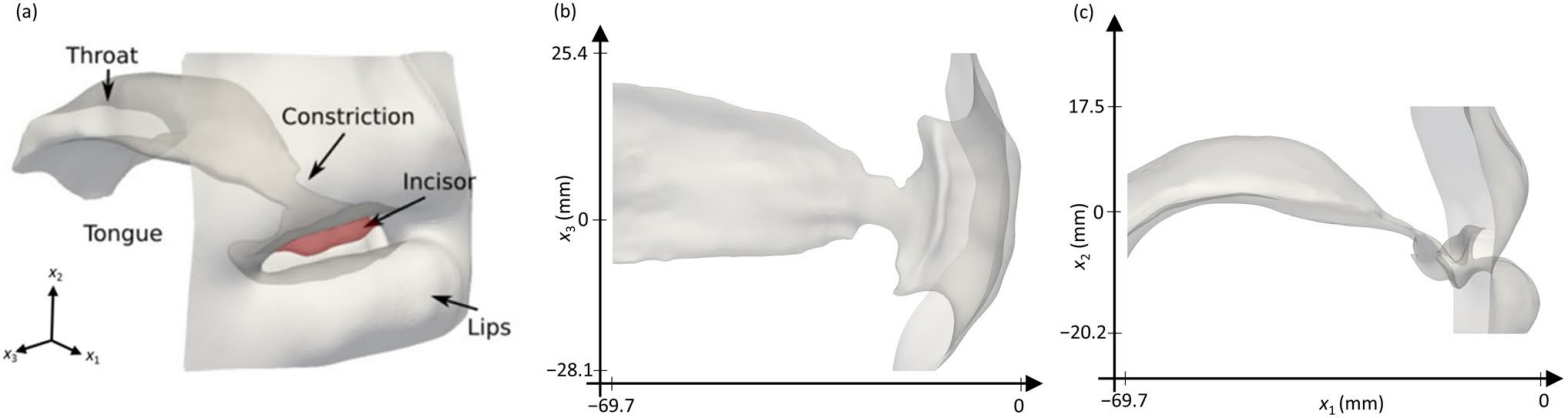
(**a**) Vocal tract geometry for pronouncing /s/. (**b**) Top and (**c**) side views of the vocal tract.

Figure 1a shows the vocal tract geometry, including the throat, tongue, hard palate, incisors, and lips. The *x*_1_ is defined as the anterior–posterior direction; the *x*_2_ is defined as the inferior–superior direction; *x*_3_ is defined as the transverse direction. The initial inclination angle of the incisor to the maxillary plane was 108° for this subject. For the inclined cases, the variation from + 10° to + 30° was resulted in 118° to 138° based on this original incisor angle, and the region of the inclination incisor is marked in red color (− 10 mm < *x*_3_ < 10 mm). The geometries from the top and side views are shown in Fig. 1b,c, respectively. We confirmed that the exclusion of the upstream vocal tract geometry on the simulation of /s/ was negligible for the main acoustic characteristics of /s/^14^. According to Runte^1^, by comparing the sound of /s/ generated by the human subject with the subject’s vocal tract replica made of plaster using a 3D printer, the frequency characteristics of /s/ were produced up to 16 kHz with the maximum discrepancy of 8 dB. It indicates that the solid wall condition is valid to investigate the sound mechanisms.

**Figure 2:**
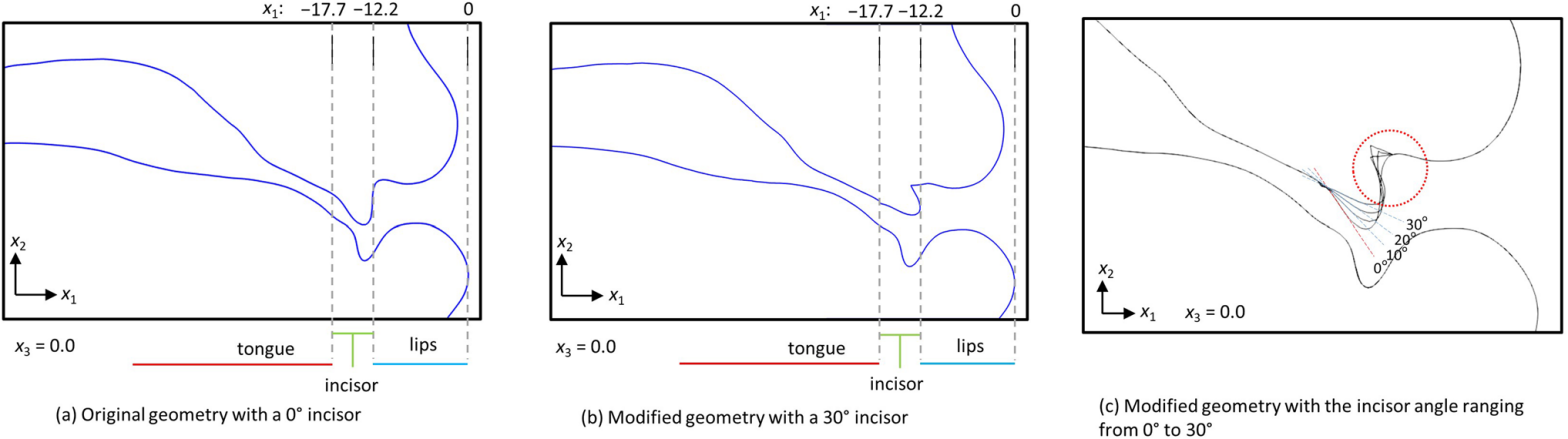
Vocal tract geometry with the incisor angle increased from the original position (0°) to 30°: (**a**) original geometry with 0°; (**b**) modified geometry with 30°; and (**c**) modified geometry with the incisor angle ranging from 0° to 30°.

To investigate the effect of the inclination angle of the upper incisor, the incisor position (− 17.7 mm < *x*_1_ <  − 12.2 mm) was raised from its original position (Fig. 2a, 0°) up to 30° (Fig. 2b). Figure 2c shows the mid-sagittal plane in the modified geometry (*x*_3_ = 0) with the incisor angle increased in 10° increments from 0° to 30°, the length of the incisor is kept as the same as the original geometry. As shown in Fig. 2a, the overjet (horizontal overlap) and overbite (vertical overlap) of the incisor angle in the original model are 2.3 mm and 0.3 mm, respectively. When the inclination angle increases, the *x*_1_ distance between the upper and lower incisors becomes longer; therefore, the overjet increases from 2.3 to 2.5 mm. Conversely, the *x*_2_ distance becomes wider; therefore, the overbite decreases from 0.3 to − 1.7 mm. These values are within the range of clinical measurements^8^. The sidewalls of each tooth were modified to a smooth appearance to prevent the formation of small gaps between the teeth that lead to the instability of flow simulation.

### Governing equations

The governing equations are the compressible Navier–Stokes equations:

1 $$ \frac{\partial U}{{\partial t}} + \frac{{\partial F_{1} }}{{\partial x_{1} }} + \frac{{\partial F_{2} }}{{\partial x_{2} }} + \frac{{\partial F_{3} }}{{\partial x_{3} }} = 0, $$

where *t* is the time, *x*_*i*_ indicates three directions in Cartesian coordinate system (*i* = 1, 2, 3), and the conservative vector *U* is

2 $$U={\left(\rho {\rho u}_{1} {\rho u}_{2} {\rho u}_{3} \rho e \right)}^{T},$$

where the flux vectors *F*_*i*_ are

3 $$ F_{i} = \left( {\begin{array}{*{20}c} {\rho u_{i} } \\ {\rho u_{i} u_{1} + P\delta_{i1} - \mu A_{i1} } \\ {\rho u_{i} u_{2} + P\delta_{i2} - \mu A_{i2} } \\ {\rho u_{i} u_{3} + P\delta_{i3} - \mu A_{i3} } \\ {\left( {\rho e + P} \right)u_{i} - \mu A_{ij} u_{j} - k\frac{\partial T}{{\partial x_{i} }}} \\ \end{array} } \right),\,\forall i = 1,2,3. $$

*ρ* is the density, *u*_*i*_ indicates the velocity components (*i* = 1, 2, 3), and *δ*_*ij*_ is the Kronecker delta.

The total energy *e* is calculated as

4 $$ e = \frac{P}{\rho (\gamma - 1)} + \frac{1}{2}\left( {u_{1}^{2} + u_{2}^{2} + u_{3}^{2} } \right), $$

and *µA*_*ij*_ is the stress term, with

5 $$ A_{ij} = \frac{{\partial u_{i} }}{{\partial x_{j} }} + \frac{{\partial u_{j} }}{{\partial x_{i} }} - \frac{2}{3}\left( {\nabla \cdot u} \right)\delta_{ij} . $$

The pressure *P* follows the ideal gas equation:

6 $$ P = \rho RT. $$

The dynamic viscosity $\mu$ and thermal conductivity *k* are based on Sutherland’s law:

7 $$ \mu \left( T \right) = \mu_{0} \left( {\frac{T}{{T_{0} }}} \right)^{\frac{3}{2}} \frac{{T_{0} + 110}}{T + 110}, $$

8 $$ k\left( T \right) = \frac{\mu \left( T \right)\gamma R}{{\left( {\gamma - 1} \right)\Pr }}, $$

where $\rho_{0}$ = 1.1842 kg/m^3^, $\mu_{0}$ = 1.85 10^−5^ N∙s/m^2^, *T*_0_ = 298.06 K, $\gamma$ = 1.4, *R* = 287 J/kg, and the Prandtl number (Pr) is 0.71.

To solve the three-dimensional compressible flow governed by Eq. (1), we applied the following numerical framework. The second-order-accurate implicit lower–upper symmetric Gauss–Seidel scheme (LUSGS) is adopted for time integration. The Roe scheme with a preconditioning method and dual time stepping is applied, and the discretized form of Eq. (1) with artificial time step Δτ is

9 $$ \Gamma \frac{{\overline{U} _{p}^{{k + 1}}  - \overline{U} _{p}^{k} }}{{\Delta \tau }} + \frac{{3\overline{U} ^{{k + 1}}  - 4\overline{U} ^{n}  + \overline{U} ^{{n - 1}} }}{{2\Delta t}} + \frac{1}{{\Delta x_{1} }}\left( {\overline{F} _{{1_{{\left( {i + \frac{1}{2},j,k} \right)}} }}^{{k + 1}}  - \overline{F} _{{1_{{\left( {i - \frac{1}{2},j,k} \right)}} }}^{{k + 1}} } \right) + \frac{1}{{\Delta x_{2} }}\left( {\overline{F} _{{2_{{\left( {i,j + \frac{1}{2},k} \right)}} }}^{{k + 1}}  - \overline{F} _{{2_{{\left( {i,j - \frac{1}{2},k} \right)}} }}^{{k + 1}} } \right) + \frac{1}{{\Delta x_{3} }}\left( {\overline{F} _{{3_{{\left( {i,j,k + 1/2} \right)}} }}^{{k + 1}}  - \overline{F} _{{3_{{\left( {i,j,k - 1/2} \right)}} }}^{{k + 1}} } \right) = 0,  $$

where *Г* is the preconditioning matrix proposed by Weiss and Smith^23^, *U*_*p*_ is the primitive form [*P*, *u*_1_, *u*_2_, *u*_3_, *T*], τ is artificial time and t is physical times, the superscripts *k* and *n* are the iteration numbers in artificial time step and the proceeding step of real time, respectively. The quantities associated with the artificial time term of the $(k + 1){\text{th}}$ iteration are transferred approximately to quantities of the $(n + 1){\text{th}}$ time step in real time when the term $\partial U_{p} /\partial \tau$ converges to zero. Then, Eq. (9) will be reduced to the original Navier–Stokes equation including the transient term.

Finally, Eq. (1) can be rearranged as

10 $$ \left[ {\frac{I}{\Delta \tau } + \Gamma^{ - 1} M\frac{3}{2\Delta t} + \Gamma^{ - 1} (\delta_{{x_{1} }} A_{p}^{k} + \delta_{{x_{2} }} B_{p}^{k} + \delta_{{x_{3} }} C_{p}^{k} )} \right]\Delta U_{p} = \Gamma^{ - 1} R^{k} , $$

where $M = \partial U/\partial U_{p}$, ${A}_{p}^{k}=\partial {F}_{1}^{k}/\partial {U}_{p}$, ${B}_{p}^{k}=\partial {F}_{2}^{k}/\partial {U}_{p}$, ${C}_{p}^{k}=\partial {F}_{3}^{k}/\partial {U}_{p}$ are the flux Jacobian, $R^{k} = - (3U^{k} - 4U^{n} + U^{n - 1} )/(2\Delta t) - (\delta_{{x_{1} }} \overline{F}_{1}^{k} + \delta_{{x_{2} }} \overline{F}_{2}^{k} + \delta_{{x_{3} }} \overline{F}_{3}^{k} )$, and $\delta_{{x_{i} }}$ is the central-difference operator.

To accelerate the convergence speed, Lian et al.^24^ proposed the solution-limited time stepping (SLTS) method by adaptively adjusting the CFL number and determine Δτ in the governing equation. When adopting LUSGS method, the estimation value Δ*Q*_*est*_ defined as

11 $$ \left| {\Delta Q_{est} } \right. = - \Delta \tau M^{ - 1} \left[ { - \left( {3U^{k} - 4U^{n} + U^{n - 1} } \right)/\left( {2\Delta t} \right) - \left( {\delta_{{x_{1} }} \overline{F}_{1}^{k} + \delta_{{x_{2} }} \overline{F}_{2}^{k} } \right)} \right], $$

and Δ*Q*_*ref*_ is defined as

12 $$ \left| {\Delta Q_{ref} } \right. = \left( {\begin{array}{*{20}c} {\alpha_{1} \times \max \left[ {0.5 \times \rho \left( {u_{1}^{2} + u_{2}^{2} } \right),\,\Delta P_{sur} ,\,P_{global} \times 10^{ - 9} } \right]} \\ {\alpha_{2} \times \max \left[ {\left( {u_{1}^{2} + u_{2}^{2} } \right),\,\frac{{\Delta P_{sur} \times c}}{\gamma P},\,V_{global} \times 10^{ - 9} } \right]} \\ {\alpha_{3} \times \max \left[ {\left( {u_{1}^{2} + u_{2}^{2} } \right),\,\frac{{\Delta P_{sur} \times c}}{\gamma P},\,V_{global} \times 10^{ - 9} } \right]} \\ {\alpha_{4} \times T} \\ \end{array} } \right), $$

where Δ*P*_*sur*_ is the maximum difference between the pressure at surrounding points and *P*_*global*_, *V*_*global*_ denotes the global value that ensures the reference values are always greater than 0, c is the speed of sound, and *γ* is the heat capacity ratio. Equation (12) provide a criterion for determining whether the calculation is stable or not. The factor [*α*_1_ *α*_2_ *α*_3_ *α*_4_] is the coefficient of the maximum allowable fractional change according to Lian^24^. Under the SLTS method, the larger physical time step and the faster speed of convergence can be achieved. However, because of the Newton linearization error of the term $\partial U_{p} /\partial \tau$, SLTS method is not suitable for aeroacoustic simulations even when the convergence criteria are satisfied. Hence, we applied the adaptively switched time stepping scheme (ASTS)^25^ to reduce the computational cost and maintain the accuracy in the aeroacoustic simulation.

In the calculation of $R^{k}$ on the right-hand side of Eq. (10), the terms involving *F*_*i*_ in Eq. (3) can be divided into an inviscid term *F*_*inviscid*_, and a viscous term *F*_*viscous*_, as shown below:

13 $$ F_{{inviscid}}  = \left( {\begin{array}{*{20}l}    {\rho u_{i} {\text{ }}}  \\    {\rho u_{i} u_{1}  + p\delta _{{i1}} }  \\    {\rho u_{i} u_{2}  + p\delta _{{i2}} }  \\    {\rho u_{i} u_{3}  + p\delta _{{i3}} }  \\    {(\rho e + p)u_{i} }  \\   \end{array} } \right), $$

14 $$  F_{{viscous}}  =  - \left( {\begin{array}{*{20}l}    0 \\    {\mu A_{{i1}} } \\    {\mu A_{{i2}} }  \\    {\mu A_{{i3}} } \\    {\mu A_{{ij}} u_{j}  + \lambda \frac{{\partial T}}{{\partial x_{i} }}}  \\   \end{array} } \right). $$

When employed the Roe scheme in Eq. (14), *F*_*inciscid*_ term will be discretized into

15 $$ F_{inviscid,i + 1/2} = \frac{1}{2}[F_{R} (U) + F_{L} (U)] + F_{d} , $$

where *F*_*d*_ is the Roe dissipation term, which is composed of jumps of properties of work fluids. For the reconstruction of *F*_*R*_ and *F*_*L*_, the fifth-order monotone upstream-centered scheme for conservation laws (MUSCL)^26^ without a limiter function to prevent turbulent fluctuations from attenuating. Aside from the inviscid term, the derivative terms in *A*_*ij*_ in the viscous term of Eq. (3) are calculated using the second-order central difference. The detail of the current framework can be found in previous study^18–20^.

### Computational conditions

To simulate the complex geometry, e.g., a realistic human oral cavity, the immersed boundary method with the hierarchical structure grid^21^ was applied for the grid spacing. As the grid configuration, the computational domain is divided according to the hierarchical structure system proposed by Nakahashi^21^. Using a hierarchical structure grid can shorten the working time required to build computational grids and simultaneously provide better load balancing and higher performance for parallel computations.

After testing the space resolution, the minimum grid size was set as 0.05 mm around the upper incisors to keep the accuracy of the immersed boundary around the turbulent region. To simulate the sound waves propagating through the lip outlet, the far-field region was set outside of the vocal tract model. The total grid numbers of the 0–30° cases were 8.7 × 10^7^, 7.2 × 10^7^, 7.8 × 10^7^, and 6.7 × 10^7^, respectively.

**Figure 3:**
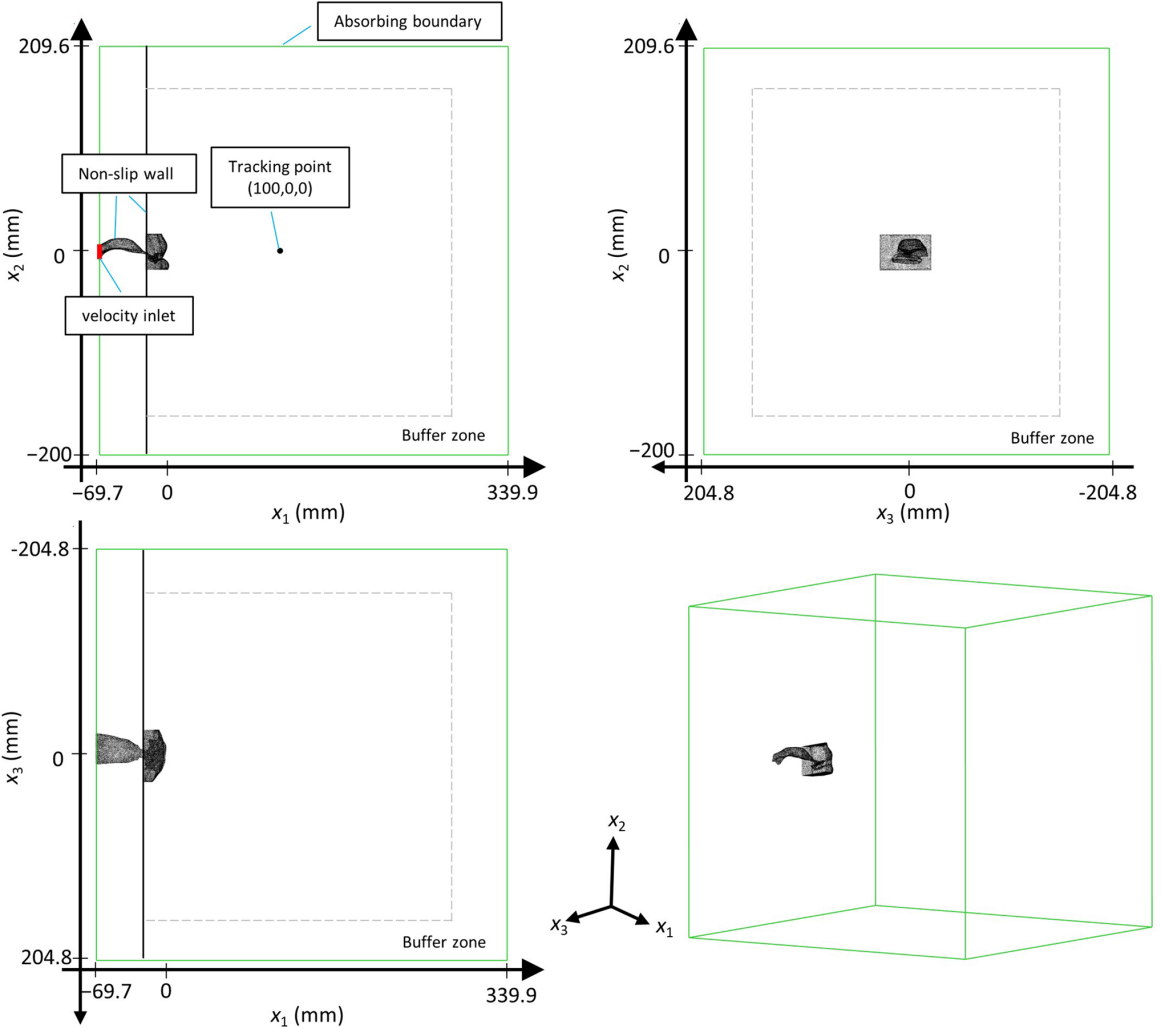
The three-view diagram of overall computational domain of the current model.

The three-view diagram of overall computational domain with the boundary condition and tracking point for the current model are shown in Fig. 3. The inlet was set to a uniform velocity condition to simulate the pronunciation of /s/. The uniform inlet velocity was set to 1.5 m/s, which resulted in a physiological flow rate of 330 cm^3^/s ^12^. The Reynolds number was 5632, based on the maximum velocity (${\stackrel{-}{\left|u\right|}}_{\mathrm{max}}=50.8\, \mathrm{m}/\mathrm{s}$) inside the oral cavity in the original geometry. To keep the flow in the computational domain from being polluted by reflecting pressure waves, an absorbing boundary condition was used as the outlet condition. The absorbing boundary condition used in the current study is based on JB Freund^27^ and extend by Li^20^ which is adjusted for the low flow speed simulation. The time step was set to 10^−6^ s, so that the CFL number was 7.8, which fulfilled the condition for the ASTS method^26^. The physical time of the performed simulations was 0.015 s and required parallel computing with 1152 cores on 32 nodes for 30 h. Table 1 summarizes the computational parameters for the simulations. Fast Fourier transform (FFT) using the Hann window was applied to the waveforms sampled 100 mm from the lip outlet (*x*_*1*_ = 100 mm) to analyze the far-field sound spectrum. The FFT sampling frequency was 50 kHz with 256 points averaged five times. The sound pressure level (SPL) was calculated based on the reference pressure *P*_ref_ = 20 × 10^−6^ Pa. In addition to the sound spectrum, the magnitudes of the velocity fluctuations at each frequency were calculated via FFT on each grid to identify the highest contribution position for the potential sound source^23^.

**Table 1:** Computational parameters of the 0° model.

Size of the largest cell (mm)	1.6
Size of the smallest cell (mm)	0.05
Computational domain (mm^3^)	409.6 × 409.6 × 409.6
Inlet velocity (m/s)	1.5
Re	5632
Time step (s)	10^−6^
Physical time (s)	0.015

## Results and discussion

**Figure 4:**
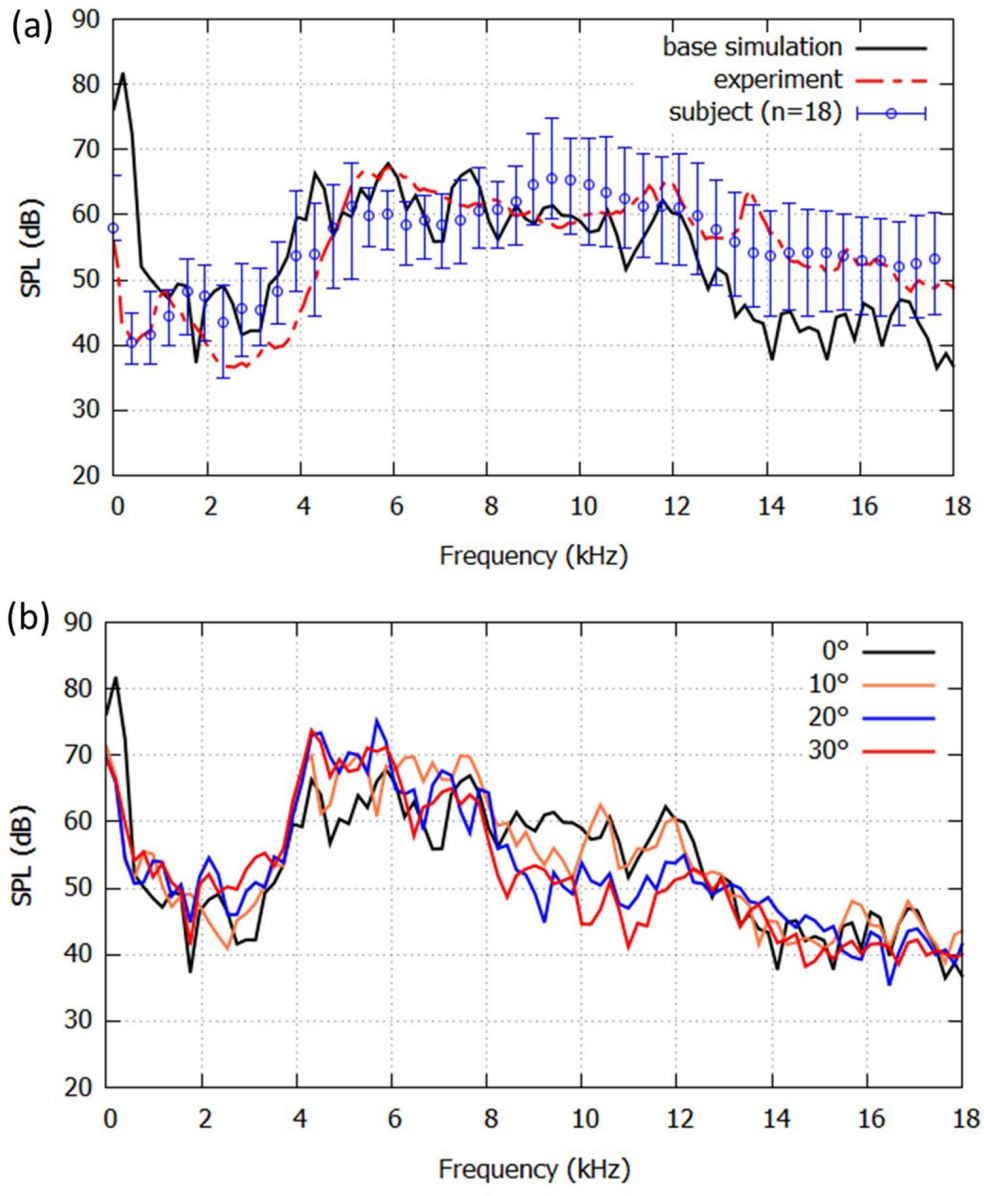
(**a**) Sound pressure level (SPL) spectrum of the base simulation (0°, solid black line) and measurements of the printed replica (dashed red line) and the average value of an actual subject for 18 recordings (blue circles). The error bars denote the range for 18 repeated recordings. (**b**) The predicted SPL spectra with incisor angles from 0° to 30°.

To ascertain the accuracy of the simulation in the present framework, the result was compared with experimental measurements. The acoustic pressure was collected 100 mm from the lip outlet (*x*_1_ = 100 mm), and the frequency spectra of the sound was calculated via FFT, as shown in Fig. 4a. We have confirmed that the velocity magnitude around the tracking point is small enough to be neglected, which is less than 0.05 m/s. In the experiment, the vocal tract replica was constructed using a 3D printer (Objet30Pro, Stratasys, USA; accuracy: ± 0.1 mm) and a constant flow was input to the model using a compressor (YC-4RS, Yaezaki, Tokyo, Japan). The sound generated by the model was measured using a microphone (type 4939, Bruel & Kjaer, Nærum, Denmark) at a distance of *x*_1_ = 100 mm in an anechoic chamber (with a volume of 8.1 m^3^). The pronunciation of /s/ was recorded with an actual subject for 18 times of utterances. The subject sustained /s/ for 3 s without vowel context, and before the signal was calculated via FFT, some portions of signals after the onset and before the offset of /s/ are removed. Therefore, inclusion of the effect of the onset and offset was avoided.

The sibilant sound is characterized as a broadband noise above 4 kHz. At a frequency of 3 kHz, SPL rapidly increased, and the first characteristic peak reached around 5 kHz. This result shows good agreement with the measurements for the actual subject and oral replica, and the same characteristics of sibilant /s/ sound was observed by Runte^1^. Accordingly, this result suggests that the present framework of the direct aeroacoustic computation can provide aeroacoustic predictions with reasonable accuracies.

To investigate the effects of the incisor angle, the incisor angle was varied from 0° to 30°; the far-field SPL spectra for these angles are shown in Fig. 4b. According to Runte^1^, the changes of incisor angle of denture led to a different noise band range. In this study, amplitudes from 8 to 12 kHz were decreased by the increase of incisor angle. This means that the upper boundary frequency of noise was decreased and the noise band range became smaller with the inclined incisors. While the noise band range of 0° was approximately 4 kHz to 12 kHz, the noise band range of 30° was 4 kHz to 8 kHz. This result is consistent with that found by Runte^1^. Besides, according to Snow^28^, general audible frequency range for male and female speech are up to 7 kHz, and 9 kHz, respectively, and the characteristic peak of the sibilant sound at around 4 kHz was observed for all teeth angles. Therefore, the sounds generated by all the cases can be characterized and recognized as sibilant fricative /s/. However, the decreasing amplitudes in the frequency range 8 kHz to 12 kHz might affect the recognition of the sound.

**Figure 5:**
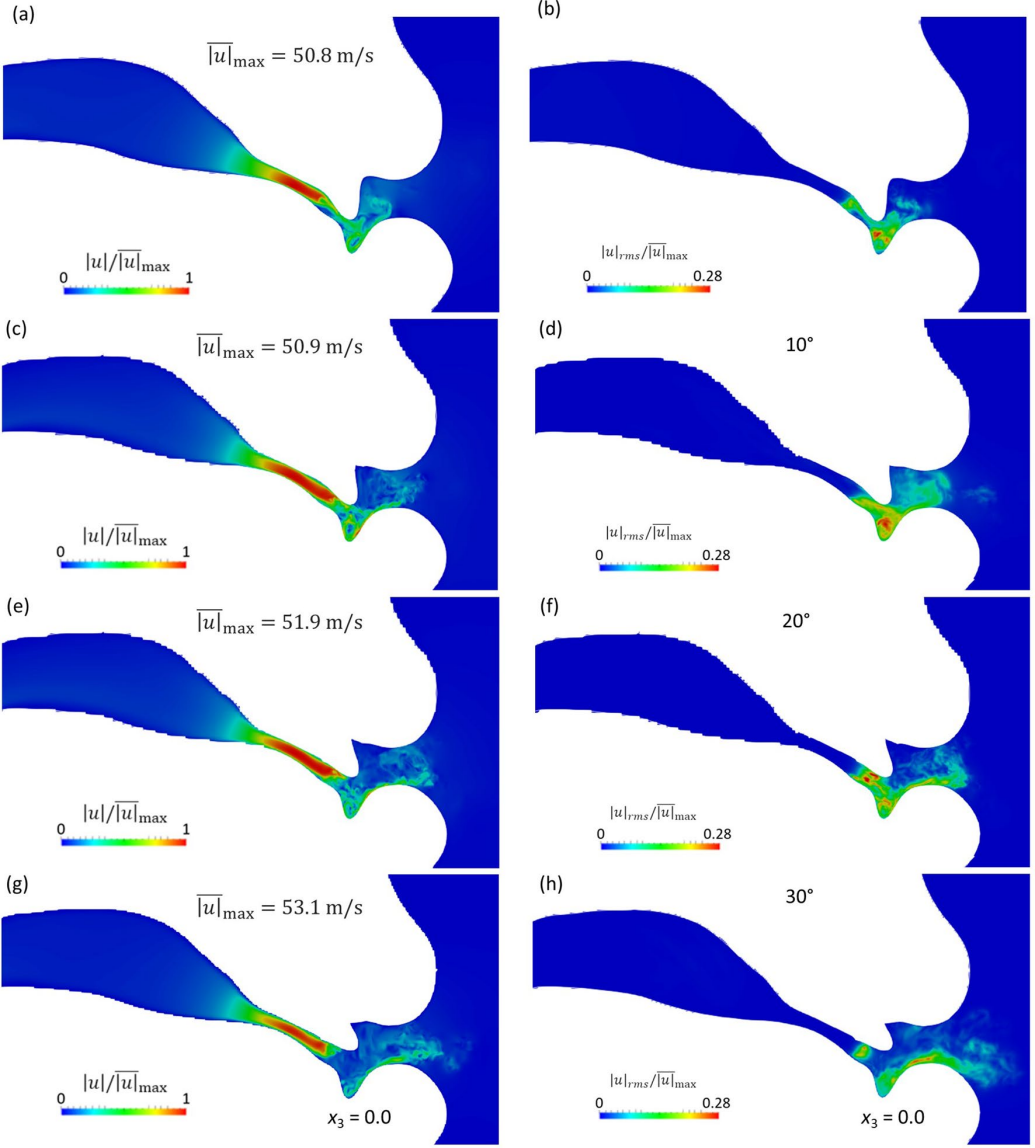
(**a,c,e,g**) Normalized instantaneous velocity magnitude and (**b,d,f,h**) root mean square (RMS) of the velocity fluctuations at t = 0.015 s for the (**a,b**) 0°, (**c,d**) 10°, (**e,f**) 20°, and (**g,h**) 30° models.

Figure 5 shows the normalized instantaneous velocity magnitude $\left|u\right|/{\stackrel{-}{\left|u\right|}}_{max}$ and root mean square (RMS) value of the velocity fluctuations ${\left|u\right|}_{rms}/{\stackrel{-}{\left|u\right|}}_{max}$ from the 0° to 30° models on the mid-sagittal plane (*x*_3_ = 0). As shown in Fig. 5a,c,e,g, the instantaneous velocities of all cases are accelerated at the narrow channel between the tongue and the hard palate (the sibilant groove) (− 25 mm < *x*_1_ <  − 16 mm). Downstream of the sibilant groove, the flow became turbulent at the region between the teeth and the lower lip (− 15 mm < *x*_1_ <  − 11 mm) as a result of the jet flow leaving the sibilant groove. However, because the exit of the sibilant groove became wider in superior direction with the inclined incisors, the reduction of the occlusion made the mainstream flow faster and the flow reached more distant positions. Hence, the high RMS region shown in Fig. 5b,d,f,h moved from the cavity between the lower incisor and the lip to the tip of the lower lip with increasing incisor angle. Meanwhile, the turbulence intensity was not affected by the raised incisor angle. According to Lighthill’s analogy^22^, the aeroacoustic sound source is primarily produced by the time variation in the space derivatives of the Reynolds stress tensor, which means that the sound source likely appeared in the region of high RMS values. Therefore, the different flow configurations caused by the raised incisor angle might be considered as the reason for the difference in the acoustic field.

**Figure 6:**
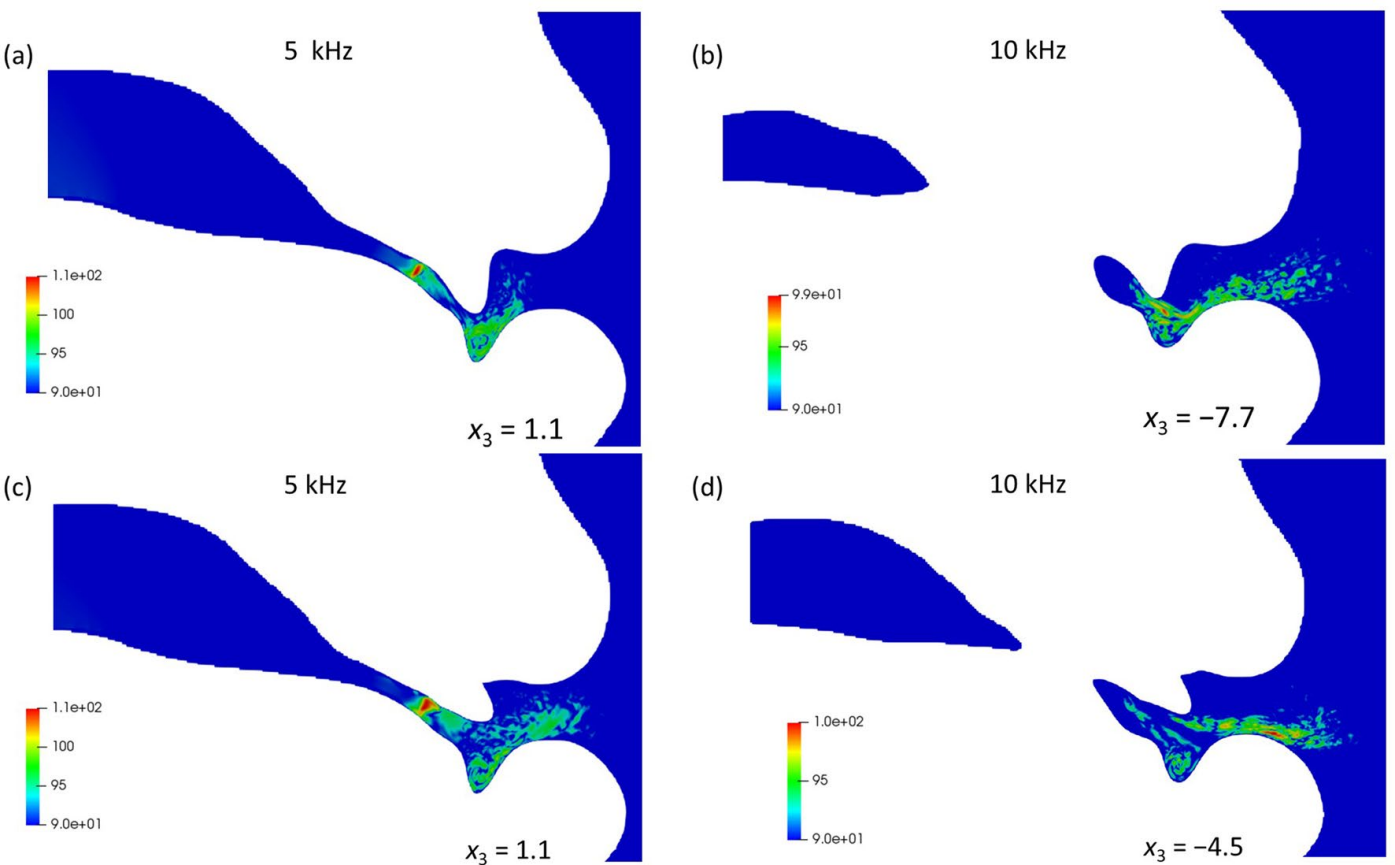
Fast Fourier transforms of the velocity fluctuations at (**a,c**) 5 kHz and (**b,d**) 10 kHz for the (**a,b**) 0° and (**c,d**) 30° models.

To identify the cause of the different SPL values around 10 kHz between 0° and 30° in Fig. 4b, the positions of the potential sound sources for the 0° and 30° models were calculated. The magnitudes of the velocity fluctuations at specific frequencies (5 kHz and 10 kHz) were calculated via FFT on each grid. The magnitudes of the velocity fluctuations inside the vocal tract for the 0° model along the *x*_1_–*x*_2_-plane are shown in Fig. 6a at 5 kHz (*x*_3_ = 1.1 mm) and Fig. 6b at 10 kHz (*x*_3_ =  − 7.7 mm), respectively. At 5 kHz, the maximum value is located behind the incisor, which is the exit of the sibilant groove. As seen in the flow configurations (Fig. 5), the jet flow emerged at this position. Conversely, the maximum value at 10 kHz appeared at the cavity between the teeth and the lower lip, which is at the position (49.1, − 8.4, − 7.7). This corresponds to the exit of the jet flow being the gap between the teeth. The magnitudes of the velocity fluctuations for the 30° model at 5 kHz (*x*_3_ = 1.1 mm) and 10 kHz (*x*_3_ =  − 4.5 mm) are shown in Fig. 6c,d. The maximum value for 5 kHz appeared at the exit of the sibilant groove, which is the same as the position for the 0° model at 5 kHz. These results indicate that the velocity fluctuations downstream of the constriction formed the characteristic peak at 5 kHz for both the 0° and 30° models. Conversely, at 10 kHz, the jet flow of the 30° model passed along the surface of the incisor and the maximum value of the velocity fluctuations appeared above the lower lip, which was at the position (58.5, − 7.7, − 4.5).

To determine the relationship between the positions of the velocity fluctuations, i.e., the assumed aeroacoustic sound sources, and the far-field SPL spectra, instead of the base simulation with constant velocity inlet from the throat, the acoustic simulations with acoustic monopole sources were conducted for the 0° and 30° cases. In the previous acoustic studies, monopole to quadrupole sound sources were used to emulate the sound generated by the turbulent flow^29,30^. Therefore, for the simplicity, the monopole sources composed of white noise were applied in the current study at Point 1 (49.1, − 8.4, − 7.7) and Point 2 (58.5, − 7.7, − 4.5), which corresponded to the positions of maximum velocity fluctuations at 10 kHz for both models.

**Figure 7:**
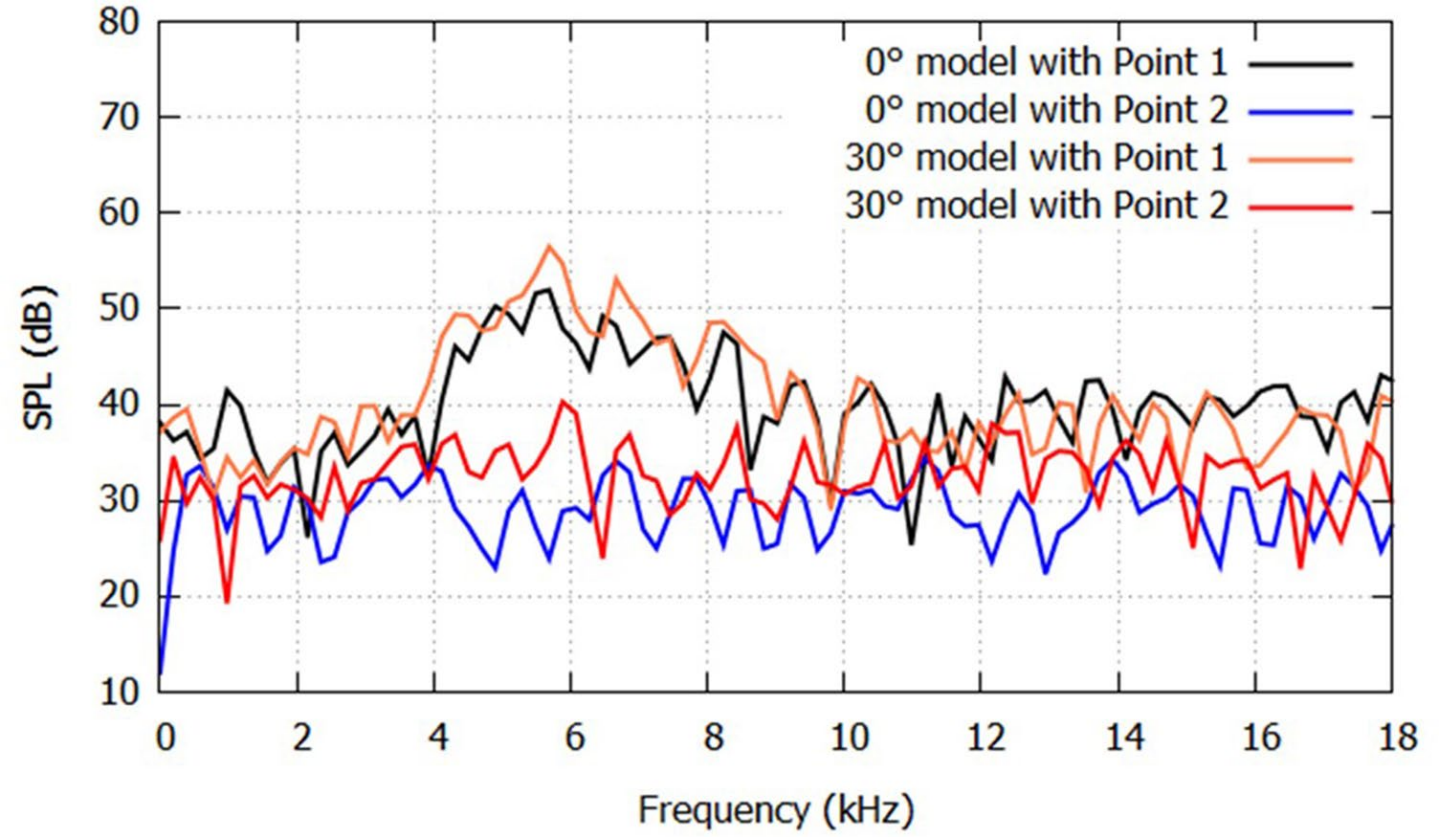
SPL spectra for the 0° and 30° models predicted for the monopole acoustic sources at Point 1 (0.049, − 0.0084, − 0.0077) and Point 2 (0.0585, − 0.0077, − 0.0045).

A comparison of the far-field sound spectra in Fig. 7 shows an obvious amplitude difference between the two sound source positions in the frequency range of 4–8 kHz. When the acoustic source is located at Point 1, SPL shows the characteristics of the sibilant sound for both the 0° and 30° models. Conversely, SPL generated by the sound source at Point 2 consisted of a broad noise with amplitudes lower than those generated at Point 1. This is because the acoustic source at Point 1 was located inside the modeled vocal tract geometry and the sound wave was resonated by the front oral cavity. The distance from the glosso-palatal constriction to anterior lip surface was 1.39 cm, which was close to the quarter of wavelength around 6 kHz. Therefore, SPL around 6 kHz was larger and the sound generated by Point 1 still had the characteristics of the sibilant sound in the far field. In contrast, because the acoustic source at Point 2 was located almost entirely outside of the vocal tract, the source did not couple strongly with the resonator, and no significant sound could be caught in the entire frequency range at the far-field. For this reason, the far-field SPL around 10 kHz was smaller in the 30° case. Consequently, the shift in the aeroacoustic source position affected the resonance of the sound waves and influenced the far-field SPLs of /s/.

These findings and the framework of the current simulation model can clarify the effects of geometrical differences resulting from dental prosthesis, e.g., the incisor positions and angles, on the flow as well as the sound generation. Besides, it can be used to help design dental prostheses at the same time predict the outcomes of surgical procedures for the production of sibilant fricatives.

## Conclusions

To investigate the effect of the inclination angle of the incisor on the speech production of the sibilant /s/, numerical flow simulations of a vocal tract geometry with different incisor angles was conducted. On the basis of the far-field SPL spectrum, increasing the incisor angle from 0° to 30° had no influence on the characteristic peak of the sibilant sound at 4 kHz. However, increasing the incisor angle reduced the amplitude of the sound in the frequency range from 8 to 12 kHz. In the flow field, the turbulence intensity kept the same level and the maximum velocity occurred at the sibilant groove in all cases, while the high RMS value region moved from the cavity between the teeth and the lower lip to the tip of the lower lip when the inclination angle increased.

By conducting acoustic simulations with a monopole source at the potential sound source positions of the 0° and 30° cases, we found that the acoustic source position affects the resonance of the sound wave and influences the far-field SPL spectrum. Specifically, if the sound source position was located closer to the exit of the vocal tract, i.e., the lips, the source didn’t couple strongly with the resonator, and no significant frequency could be caught at far-field. Because the flow channel downstream of the sibilant groove became wider when the incisor angle was increasing from 0° to 30°, the large velocity fluctuation region was shifted and the amplitude of the far-field sound around 10 kHz was reduced. Consequently, the slight change of the geometry affected less to the turbulent intensity, but changed the flow configuration and shifted the position of the potential sound source, thereby influenced the performance on the acoustics field. These results provide the underlying insights necessary to design dental prostheses for the production of sibilant fricatives.

## Data Availability

The datasets generated during and/or analysed during the current study are available from the corresponding author on reasonable request.
